# Codon 89 polymorphism in the human 5 **α** -reductase gene in primary breast cancer

**DOI:** 10.1054/bjoc.2000.1681

**Published:** 2001-03

**Authors:** A Scorilas, B Bharaj, M Giai, E P Diamandis

**Affiliations:** Department of Pathology and Laboratory Medicine, Mount Sinai Hospital and Department of Laboratory Medicine and Pathobiology, University of Toronto, Toronto, Ontario, M5G 1X5, Canada; National Center for Scientific Research ‘Demokritos’, Athens, 153 10, Greece; Department of Gynecologic Oncology, Institute of Obstetrics and Gynecology, University of Turin, Turin, 10128, Italy

**Keywords:** point mutations, polymorphisms, 5α-reductase, androgens and breast cancer, breast cancer prognosis, hormones and cancer, SRD5A2, breast cancer, PSA, androgens

## Abstract

The enzyme human steroid 5-α reductase type II (SRD5A2) and androgen receptor (AR) are critical mediators of androgen action, suggesting a potential role in hormonally related cancers. The SRD5A2 gene harbours two frequent polymorphic sites, one in the coding region, at codon 89 of exon 1, where valine is substituted by leucine (V89L) and the other in the 3′ untranslated region (3′ UTR) where a variable number of dinucleotide TA repeat lengths exists. The V89L polymorphism is known to alter the activity of this enzyme. In the present study we examined 144 sporadic breast tumours from Italian patients for the V89L and TA polymorphisms by sequence and fragment analysis, respectively. Tumour extract prostate specific antigen (PSA) concentration as well as a number of well-established clinical and pathological parameters were evaluated. The results show that 53% of the tumours were homozygous for VV alleles, 37% were heterozygous for VL alleles and 10% were homozygous for LL alleles. TA(0) repeats were found in tumours with VV, LL and VL genotypes. TA(9) repeats were only found in VV homozygotes and were totally absent from either LL homozygotes or VL heterozygotes. PSA expression was significantly elevated in tumours with VV genotype. The presence of LL alleles in breast tumours is associated with earlier onset and shorter disease-free (RR = 2.65;*P* = 0.013) and overall survival (RR = 3.06;*P* = 0.014) rates. The VV genotype is associated with a more favourable prognosis. Our study suggests that the polymorphism in codon 89 of exon 1 of the human 5α-reductase gene is related with TA repeat genotypes, PSA expression and breast cancer prognosis. More specifically, we found that the LL genotype is also associated with earlier onset and more aggressive forms of breast cancer. Long-term-outcome studies are needed to investigate the relevance of this polymorphism to breast cancer susceptibility. © 2001 Cancer Research Campaign http://www.bjcancer.com


*British Journal of Cancer* (2001) **84**(6), 760–767
© 2001 Cancer Research Campaign
doi: 10.1054/ bjoc.2000.1681, available online at http://www.idealibrary.com on IDEAL®

http://www.bjcancer.com

### Codon 89 polymorphism in the human 5α-reductase gene in primary breast cancer

**A Scorilas[1,2], B Bharaj[1], M Giai[3] and EP Diamandis[1]**

[1]Department of Pathology and Laboratory Medicine, Mount Sinai Hospital and Department of Laboratory Medicine and Pathobiology, University of Toronto, Toronto, Ontario M5G 1X5, Canada; [2]National Center for Scientific Research 'Demokritos', Athens 153 10, Greece; [3]Department of Gynecologic Oncology, Institute of Obstetrics and Gynecology, University of Turin, Turin, 10128, Italy

**Summary** The enzyme human steroid 5-α reductase type II (SRD5A2) and androgen receptor (AR) are critical mediators of androgen action, suggesting a potential role in hormonally related cancers. The SRD5A2 gene harbours two frequent polymorphic sites, one in the coding region, at codon 89 of exon 1, where valine is substituted by leucine (V89L) and the other in the 3′ untranslated region (3′ UTR) where a variable number of dinucleotide TA repeat lengths exists. The V89L polymorphism is known to alter the activity of this enzyme. In the present study we examined 144 sporadic breast tumours from Italian patients for the V89L and TA polymorphisms by sequence and fragment analysis, respectively. Tumour extract prostate specific antigen (PSA) concentration as well as a number of well-established clinical and pathological parameters were evaluated. The results show that 53% of the tumours were homozygous for VV alleles, 37% were heterozygous for VL alleles and 10% were homozygous for LL alleles. TA(0) repeats were found in tumours with VV, LL and VL genotypes. TA(9) repeats were only found in VV homozygotes and were totally absent from either LL homozygotes or VL heterozygotes. PSA expression was significantly elevated in tumours with VV genotype. The presence of LL alleles in breast tumours is associated with earlier onset and shorter disease-free (RR = 2.65; $P = 0.013$) and overall survival (RR = 3.06; $P = 0.014$) rates. The VV genotype is associated with a more favourable prognosis. Our study suggests that the polymorphism in codon 89 of exon 1 of the human 5α-reductase gene is related with TA repeat genotypes, PSA expression and breast cancer prognosis. More specifically, we found that the LL genotype is also associated with earlier onset and more aggressive forms of breast cancer. Long-term-outcome studies are needed to investigate the relevance of this polymorphism to breast cancer susceptibility. © 2001 Cancer Research Campaign http://www.bjcancer.com

**Keywords**: point mutations; polymorphisms; 5α-reductase; androgens and breast cancer; breast cancer prognosis; hormones and cancer, SRD5A2, breast cancer, PSA, androgens

Breast cancer annually afflicts over half a million women (Pisani et al, 1999). Androgens play an important role in the development and progression of breast cancer (Secreto et al, 1983; Oriana et al, 1987). Increased serum levels of androgens correlate with an increased likelihood of disease (Grattarola, 1973; Grattarola et al, 1974; Key and Pike, 1988). Nearly 60% of women with breast cancer show some degree of hypertestosteronaemia (Secreto et al, 1983). Excessive production of testosterone has also been reported in women with atypical breast duct hyperplasia, a precursor of breast cancer (Stoll and Secreto, 1992). Androgen receptor (AR) has been detected in almost 90% of breast cancer specimens (Bryan et al, 1984; Soreide et al, 1992) and has been shown to be a favourable prognostic factor for disease-free survival (Kuenen-Boumeester et al, 1996). The AR status and levels in breast cancer cells are positively correlated with the expression of a number of proteins, namely prostate-specific antigen (PSA) (Hall et al, 1998) and pepsinogen C (Balbin and Lopez. Otin, 1996) which are both up-regulated by androgens and act as markers of cell differentiation and favourable prognosis (Vozoso et al, 1995; Hahnel and Hahnel 1996; Scorilas et al, 1999). Androgens have also been detected in breast duct fluid (Hill et al, 1983) and in normal and cancerous tissue (Vermeulen et al, 1986; Recchhione et al, 1995; Secreto et al, 1996).

The enzyme human steroid 5-α reductase type II (SRD5A2) and AR are critical mediators of androgen action. Two isoforms of steroid 5-α reductase are known: type I enzyme encoded by the SRD5A1 gene, which is expressed mostly in the newborn scalp, skin and liver. The type II enzyme which is primarily expressed in genital skin and prostate is encoded by the SRD5A2 gene (Wigley et al, 1994). This enzyme reduces testosterone to its more potent form dihydrotestosterone (DHT) in the presence of NADPH (nicotine adenine dinucleotide phosphate; reduced form) as a cofactor (Coffey, 1993). DHT binds to the AR and the DHT-AR complex transactivates a number of genes with AR-responsive elements in their promoter sequences. One such gene is PSA. In females, breast is the major tissue capable of producing PSA. In-vitro studies have shown that PSA production in breast tissue is up-regulated by androgens and progestins (Zarghami et al, 1997). Relatively high amounts of PSA protein in breast cancer tissues are associated with steroid hormone receptor positivity, early disease stage, and other pathological and clinical features of favourable prognosis (Yu et al, 1995, 1996, 1998; Griniatsos et al, 1998).

Although to-date more than 25 point mutations have been reported in the 5 exons of the SRD5A2, only 2 of these, the codon 89 valine to leucine (V89L) and codon 49 alanine to threonine

*Received 1 September 2000*
*Revised 11 December 2000*
*Accepted 19 December 2000*

*Correspondence to:* P Diamandis substitution (A49T) along with the variable length dinucleotide TA repeats on the 3′ untranslated region of this gene occur sufficiently frequently in the population to be considered as gene polymorphisms (Vilchis et al, 1997). These alterations are capable of affecting the incidence of prostate cancer (Reichardt et al, 1995). These 2 single nucleotide changes as well as other mutations, as shown by site-directed mutagenesis, impair the reductase activity in one way or the other (Wigley et al, 1994; Makridakis et al, 1997; Makridakis et al, 2000). Decrease in the activity of this enzyme in prostate cancer has been linked to favourable prognosis (Kantoff et al, 1997). The decrease in prostate volume and PSA level in men treated with the reductase inhibitor finasteride, lends more credence to this idea (Gormley et al, 1992; Magklara et al, 1999, 2000; Scorilas et al, 2000). No data is available on the role of this enzyme in breast cancer. In prostate cancer, the homozygote VV genotype of the V89L polymorphism has been associated with high reductase activity and high DHT levels. The homozygote LL genotype has been associated with low reductase activity and low DHT levels. The heterozygote VL status has been linked to intermediate levels based upon studies on various ethnic groups (Makridakis et al, 1997). Longer dinucleotide TA repeats have been thought to decrease the reductase activity and thereby decrease the DHT levels and PSA respectively, in prostate cancer (Kantoff et al, 1997). No statisticly significant difference was found between the distribution of the TA genotypes in the breast cancer and without breast cancer women (Bharaj et al, 2000). The frequency of the V89L polymorphism in healthy females is the same as in healthy males (Vilchis et al, 1997). No data is available on the implications of these polymorphisms and 5-α-reductase activity in breast cancer. We hypothesized that there is an androgen-mediated contribution to the aetiology of breast cancer and that polymorphisms in effectors and target genes of the androgen pathway have the potential to influence breast cancer development and prognosis. Therefore, we undertook this investigation to examine the role of the V89L polymorphism in breast cancer prognosis.

### SUBJECTS AND METHODS

### Subjects

Tumour specimens were obtained from 151 breast cancer patients. The patients were undergoing surgical treatment for primary breast carcinoma at the Department of Gynecologic Oncology at the University of Turin, Italy during the period from January 1988 to December 1992. Tumour tissue had been frozen in liquid nitrogen immediately after surgery. The selection criteria for the specimens included the availability of sufficient tissue mass for extraction and assay; the patients represented 60% of new cases of breast cancer diagnosed and treated at the above institution during the accrual period. This study had been approved by the Institutional Review Board of the University of Turin.

The median age of the patients was 54 years, with a range of 25 to 93 years. All patients had a histologically confirmed diagnosis of primary breast cancer and received no treatment before surgery. Modified radical mastectomy with axillary lymph node dissection was performed on 95% of the patients. For the patients who had axillary node dissection, the positivity rate for cancer involvement of lymph nodes was 62%. The sizes of the tumours ranged from 0.8 to 7.0 cm and the mean and median sizes were 2.7 cm and 2.5 cm, respectively. Pathologic staging was performed according to the Postsurgical International Union Against Cancer Tumor-Node-Metastasis classification system (Spiessl et al, 1989). Of 150 patients for whom the stage was known, 45 (30.0%), 87 (58.0%), 7 (4.7%) and 11 (7.3%) had stages I, II, III and IV, respectively. Histologic grade of the tumours was determined according to criteria reported by Bloom and Richardson (1957), and was known for 107 patients: 6 patients (5.6%) had grade I, 57 (53.3%) had grade II and 44 patients (41.1%) had grade III. Most of the tumours (70.2%) were of invasive ductal histologic type, whereas the remaining tumours were invasive lobular (12.6%), ductal in-situ (2.0%), medullary (2.6%), papillary (2.6%), tubular (2.0%), inflammatory (2.6%), tubulo-lobular (1.3%), cribriform (2.6%), Paget (0.7%) and muciparous (0.7%). Post-operative treatment was known for all patients. Whereas 29% received no further treatment after tumour resection, 25% were given adjuvant chemotherapy only, 41% were treated with endocrine therapy only and 5% were given both chemotherapy and endocrine therapy. Disease relapse was defined as the first documented evidence of local or regional axillary recurrence or distant metastasis.

Information of follow-up was available for all patients and included survival status (alive or deceased) and disease status (disease-free or recurrence/metastasis) along with the dates of the events and cause of death, if applicable. The relapse-free survival time in each case was the time interval between the date of surgical removal of the primary cancer and the date of the first documented evidence of relapse. The overall survival time was the time interval between the date of surgery and the date of death, or the date of last follow-up for those who were alive at the end of the study. During their respective follow-up periods, 56 patients (37.1%) developed cancer relapse and 39 (25.8%) died.

### Extraction of DNA

DNA was extracted from tissues using the Qiagen tissue DNA extraction kit (Qiagen, Chatsworth, CA, USA). Approximately 25 mg of tissue was used to extract the DNA. The breast tissue which contained more than 70% tumour cells, as determined by histological examination, was pulverized into a fine powder and stored until used at −80˚C. Briefly, after the lysis of cells, the DNA was entrapped onto the silica membrane, washed and eluted in a buffer solution. DNA was quantified by absorbance measurements at 260 nm and stored at 4˚C until analysis.

### Amplification of the V89L polymorphism region by PCR

Two paired primer sequences flanking the V89L polymorphism region were used and their sequences were as follows: 5′-GCA GCG GCC ACC GGC GAG G-3′ and 5′-AGC AGG GCA GTG CGC TGC ACT-3′.

The oligonucleotides were designed using the computer software Oligo 5.0 (National Biosciences Inc, Plymouth, MN), according to the SRD5A2 gene sequence deposited in Genebank by Labrie et al (1992), accession # L03843. PCR amplification was performed in a final volume of 25 μl, containing approximately 100–150 ng of template DNA, 10 mM PCR buffer, 2.5 units of Taq polymerase (Roche Molecular Systems), 250 μM of deoxynucleoside triphosphates, 2.25 mM $MgCl_2$ and 1 μM of each primer. The thermal cycling consisted of a 30 s denaturation at 94˚C, annealing at 65˚C for 30 s and extension at 68˚C for 1 min, and it was repeated for 30 cycles. The final extension was at 68˚C for 7 min. The PCR was initiated by a 5 min denaturation at 95˚C.

© 2001 Cancer Research Campaign 

The success of PCR was verified by running 8 μl of the amplified product on a 2% agarose gel containing ethidium bromide.

### DNA sequencing

The PCR product was sequenced on a Visible Genetics automated sequencing apparatus (Visible Genetic Inc; Toronto, Canada). The sequencing primers were labelled at the 5′-end with the fluorescent dye Cy 5.5. The sequencing primers used were as follows: 5′-GCA ACG AGC ACA CGG AGA GC-3′ and 5′-GCA GGG CAG TGC GCT GCA CT-3′. The sequencing protocol and the cycle sequencing conditions used were as follows: Initial denaturation for 5 min at 95˚C, then cycling denaturation at 94˚C for 1 min; annealing/extension at 65˚C for 1 min, for 35 cycles, followed by a final extension for 5 min at 65˚C. The sequencing was carried out as previously described (Bharaj et al, 1998). Both strands were sequenced.

### Fragment analysis of dTA repeat lengths

The TA repeat lengths were PCR-amplified using fluorescently labelled Cy 5.5 primers. Fragment analysis was carried out on a Visible Genetics Automated DNA Sequencer as previously described (Bharaj et al, 1999).

### Steroid hormone receptor analyses

Tumour specimens ($n$ = 148) were pulverized in liquid nitrogen, homogenized in buffer, and the cytosolic fractions were obtained by ultracentrifugation and quantified for steroid hormone receptors as described elsewhere (Dressler et al, 1988). The results of the dual ligand-binding assay, in which dextran-coated charcoal was used to separate bound from free ligand, were interpreted by Scatchard analysis (Scatchard, 1949). Protein concentrations of the cytosols were determined by the Lowry method (Lowry et al, 1951). Tumours with ER and PR concentrations below or equal to 10 fmol $mg^{-1}$ protein were considered as receptor negative, whereas tumours with receptor concentrations above such values were considered positive, as followed previously (Reiher et al, 1987; Alexieva-Figusch et al, 1988). Based on these cutoffs, 99 (67.3%) and 93 (63.7%) of 147 and 146 breast carcinomas were ER- and PR-positive, respectively.

### PSA immunoassay

The determination of total PSA concentration in all samples was performed by an ultrasensitive time-resolved immunofluorometric assay as described elsewhere (Ferguson et al, 1996). The PSA assay has a detection limit of 0.001 ng $ml^{-1}$. All specimens were measured in duplicate. The values used for statistical analysis were adjusted for total protein content and are expressed as ng of PSA per g of total protein in the cytosolic extracts.

### Statistical analysis

Associations between V89L genotypes and other categorical variables were analysed using the chi-square ($\chi^2$) test. ER and PR values were categorized into positive and negative status as described above. The cutoff value for tumour size was 2 cm. Lymph node status was either positive (histological evidence of tumour extension to one or more lymph nodes) or negative. Age was categorized into three groups: less than 45 years, 45 to 55 years and greater than 55 years. Kruskal-Wallis test was used for statistical evaluation of differences in PSA values between 3 groups of V89L genotypes. In this analysis, PSA was used as a continuous variable. Survival analyses were performed by constructing Kaplan–Meier DFS and OS curves (Kaplan and Meier, 1958), whereas differences between curves were evaluated by the log-rank test. Cox regression analyses using the SAS statistical software (SAS Institute, Cary, NC) was used to calculate the RR and 95% CI. Only patients for whom the status of all variables was known were included in the multivariate models, which incorporated V89L genotypes and all other variables for which the patients were characterized.

### RESULTS

### Distribution of V89L alleles and their relationship with TA length repeats

Figure 1 represents sequencing chromatograms of the polymorphism in the SRD5A2 gene, showing the base change at codon 89. The sequencing was carried out in 144 breast tumours. Among these tumours, 77 (53.5%) had GTA nucleotides at codon 89 (VV alleles), 53 (36.8%) were heterozygous with GTA/CTA codons (VL alleles) and the remaining 14 (9.3%) were homozygous for CTA codon (LL alleles). No other point mutations, insertions, or deletions were detected in the DNA sequences flanking the codon 89 region of the SRD5A2 gene. The distribution of TA repeat lengths in the same DNAs is shown in Table 1.

Table 1 also shows the relationship between V89L genotypes and the dinucleotide TA repeat lengths: TA(0), no repeats present; TA(9), 9 TA repeats. It is evident that 70% of the TA(0)/TA(9) heterozygotes are associated with homozygous VV allele, 30% with VL allele and none with the homozygous LL allele. All TA(9) homozygotes are associated with VV allele. All tumours with LL allele have TA(0) genotype. All these differences are statistically significant ($P$ < 0.001). TA(0) repeats were only found in VV, LL homozygotes as well as in VL heterozygotes. TA(9) repeat lengths were only found in VV homozygotes and were totally absent in the LL homozygotes. Thus, the TA(9) repeat appears to be linked to the VV genotype. Absence of (TA)9-LL genotype might indicate linkage disequilibrium.

### Associations of V89L genotypes to other prognostic variables

The distributions of V89L genotypes – VV, VL and LL – between subgroups of patients differing by age, tumour size, nodal status, grade, histological type, disease stage, ER status, PR status and adjuvant treatment administered were examined by the chi-square test (Table 2). LL genotype was found more frequently in younger

**Table 1** Relationship between the V89L and TA polymorphisms of the 5α-reductase gene

| TA genotypes | V89L genotypes (%) | | | | *P* value |
|---|---|---|---|---|---|
| | VV | VL | LL | Total | |
| TA(0) | 42 (43.3) | 41 (42.3) | 14 (14.5) | 97 | |
| TA(0)/(9) | 28 (70.0) | 12 (30.0) | 0(0.0) | 40 | $P$ < 0.001 |
| TA(9) | 7 (100.0) | 0 (0.0) | 0 (0.0) | 7 | |
| Total | 77 | 53 | 14 | 144 | |

 © 2001 Cancer Research Campaign

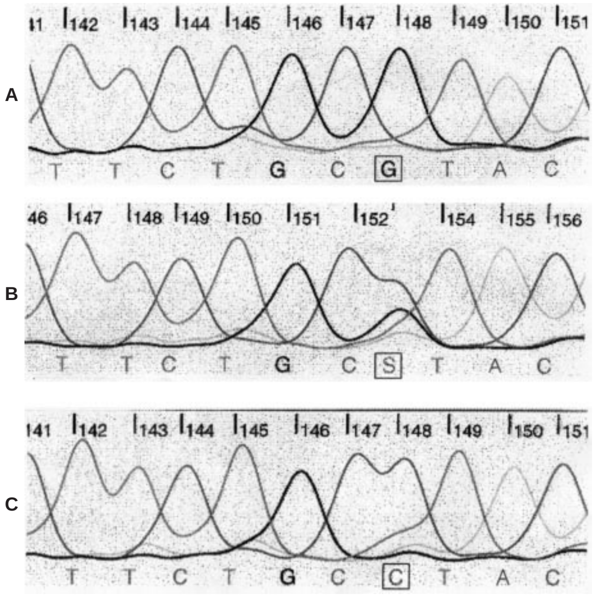

**Figure 1** Sequence analysis of SRD5A2 gene. Exon 1 was amplified from genomic DNA by PCR with primers 1F (5′-GCA ACG AGC ACA CGG AGA GC- 3′) and 1R (5′-GCA GGG CAG TGC GCT GCA CT-3′). The sequencing primers were labelled at the 5′-end with the fluorescent dye Cy 5.5. The amplified fragments were subjected to nucleotide sequence with visible genetics automated sequencing machine (Visible Genetic Inc; Toronto, Canada). The chromatograms A, B and C show the V89V (GTA homozygous), V89L (GTA/CTA heterozygous) and L89L (CTA homozygous) respectively

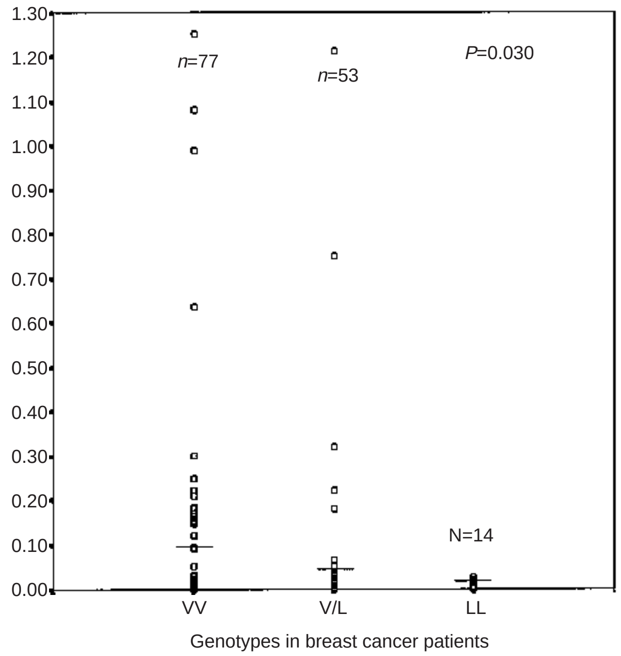

**Figure 2** Relationship between V89L genotypes and breast tumour extract PSA concentrations. The median values were 0.099 ng mg$^{-1}$, 0.076 ng mg$^{-1}$ and 0.036 ng mg$^{-1}$ protein for VV, VL and LL genotypes, respectively; *P* = 0.030, as determined by the Kruskal-Wallis test patients (below 45 years) as well as in grade III patients ($P$ = 0.008 and $P$ = 0.037 respectively). Differences in tumour tissue PSA concentrations between the V89L genotypes were found to be statistically significant by the Kruskal-Wallis test ($P$ = 0.030) (Figure 2). Statistically significant associations between V89L genotype and tumour size, nodal status, stage, histological type and steroid hormone receptors were not observed. In the same analysis, V89L genotypes were shown not to differ between patients who received different post-operative treatment modalities.

### *V89L polymorphism and breast cancer survival*

Univariate and multivariate Cox regression models were developed to evaluate the effect of V89L genotypes on DFS and OS for breast cancer patients (Table 3). These regression models demonstrated an increase in risk for relapse and death in patients with the LL genotype compared to those with the VV or VL genotype. The unfavourable DFS and OS rate of LL patients relative to those of VV or VL patients is also shown by Kaplan–Meier survival analysis (Figure 3). In the multivariate analysis of V89L genotypes, the Cox regression models were adjusted for age, nodal status, tumour size, and ER and PR status, all of which were used as categorical variables, except tumour size, which was used as a continuous variable.

### DISCUSSION

There is evidence that androgens play a role in the development and progression of breast cancer. The product of the 5-α-reductase activity, DHT, is a more potent androgen, with higher affinity for the AR than its precursor, TT. DHT acts as a mitogen and can bind to the AR and transactivate a number of androgen responsive genes (Coffey, 1993). SRD5A2 is one such gene that is regulated by androgens.

A prevalent polymorphism of a valine to leucine substitution at codon 89 in exon 1 of the SRD5A2 gene has been reported in males and females (Vilchis et al, 1997). The frequency of this polymorphism is not different between males and females. This V89L substitution has been reported to influence the activity of the reductase enzyme (Wigley et al, 1994). In this study, we examined this polymorphism in DNA from breast tumours. The most common allele was homozygous valine (GTA) (53.5%), followed by heterozygous valine/leucine (GTA/CTA) (37%); the remaining patients were homozygous for leucine (CTA) (10%). No other substitution flanking this polymorphism was detected in any of the breast tumours, including the codon 91 substitution of thymine (TAC) for guanine (GAC), as reported by Wilson et al (1993). Since the V89L polymorphism alters the coding region of the protein, in-vitro kinetic studies (Ross et al, 1992) have shown that the leucine variant (LL) decreases the activity of the 5-α reductase enzyme by almost a third, in comparison to its valine counterpart (VV). This relationship holds in-vivo too and affects PSA expression (Makridakis et al, 1997). Makridakis et al investigated the biochemical and pharmacogenetic dissection of the SRD5A2 enzyme by analysing 10 missense substitutions and 3 double mutations, all of which are naturally found in humans. It was reported that all except one of these mutations are capable of significantly influencing the enzyme activity (Makridakis et al, 2000).

The results shown in Figure 2 indicate differences in PSA concentration of breast cytosolic extracts in patients with VV and

*© 2001 Cancer Research Campaign* 

**Table 2** Relationship between V89L genotypes and clinical/pathological features of breast cancer

| Features | Total | No. of patients (%) VV | VL | LL | *P* value[a] |
|---|---|---|---|---|---|
| Age (years) | | | | | |
| <45 | 38 | 18 (50.0) | 9 (25.0) | 9 (25.0) | 0.008 |
| 45–55 | 38 | 18 (51.4) | 15 (42.9) | 2 (5.7) | |
| >55 | 75 | 41 (56.2) | 29 (39.7) | 3 (4.1) | |
| Tumour size (cm) | | | | | |
| <2 | 43 | 20 (48.8) | 18 (43.9) | 3 (7.3) | 0.51 |
| ≥2 | 105 | 55 (55.0) | 34 (34.0) | 11 (11.0) | |
| Nodal status | | | | | |
| Negative | 55 | 28 (53.8) | 20 (38.5) | 4 (7.7) | 0.85 |
| Positive | 88 | 45 (52.9) | 31 (36.5) | 9 (10.6) | |
| Grade[b] | | | | | |
| I–II | 63 | 36 (59.0) | 22 (36.1) | 3 (6.6) | 0.037 |
| III | 44 | 23 (53.5) | 11 (26.2) | 9 (16.7) | |
| Histology | | | | | |
| Ductal | 106 | 58 (57.4) | 32 (31.7) | 11 (10.9) | 0.228 |
| Lobular | 19 | 9 (50.0) | 9 (50.0) | 0 (0.0) | |
| Other | 26 | 10 (40.0) | 12 (48.0) | 3 (12.0) | |
| Stage[c] | | | | | |
| I | 45 | 25 (58.1) | 13 (30.2) | 5 (11.6) | 0.69 |
| II | 87 | 41 (50.0) | 34 (41.5) | 7 (8.5) | |
| III–IV | 18 | 11 (61.1) | 5 (27.8) | 2 (11.1) | |
| ER status[d] | | | | | |
| Negative | 48 | 25 (54.3) | 15 (32.6) | 6 (13.0) | 0.49 |
| Positive | 99 | 50 (53.2) | 37 (39.4) | 7 (7.4) | |
| PR status[d] | | | | | |
| Negative | 53 | 30 (61.2) | 16 (32.7) | 3 (6.1) | 0.33 |
| Positive | 93 | 44 (48.9) | 36 (40.0) | 10 (11.1) | |
| Adjuvant treatment | | | | | |
| None | 45 | 22 (51.2) | 16 (37.2) | 5 (11.6) | 0.09 |
| Tamoxifen | 62 | 31 (51.7) | 27 (45.0) | 2 (3.3) | |
| Chemotherapy ± tamoxifen | 44 | 24 (58.5) | 10 (24.4) | 7 (17.1) | |

[a] $\chi^2$ test. [b] Bloom-Richardson grading system. [c] TNM system. [d] Cutoff point: 10 fmol $mg^{-1}$ protein.

**Table 3** Association between V89L genotypes and breast cancer survival

| Variable | Disease-free survival RR[a] (95% CI)[b] | *P* value | Overall survival RR[a](95% CI)[b] | *P* value |
|---|---|---|---|---|
| Univariate analysis | | | | |
| VV | 1.00 | | 1.00 | |
| VL | 1.15 (0.64–2.09) | 0.62 | 1.18 (0.56–2.46) | 0.65 |
| LL | 2.65 (1.23–5.71) | 0.013 | 3.06 (1.25–7.45) | 0.014 |
| Multivariate analysis[c] | | | | |
| VV | 1.00 | | 1.00 | |
| VL | 1.04 (0.55–1.96) | 0.90 | 1.09 (0.49–2.43) | 0.81 |
| LL | 1.91 (0.79–4.58) | 0.14 | 2.55 (0.90–7.27) | 0.078 |

[a] Relative risk (RR) estimated from Cox proportional hazard regression model. [b] Confidence interval of the estimated RR. [c] Multivariate models were adjusted for lymph nodes status; tumour size; patient age; ER and PR expression.

 *© 2001 Cancer Research Campaign*

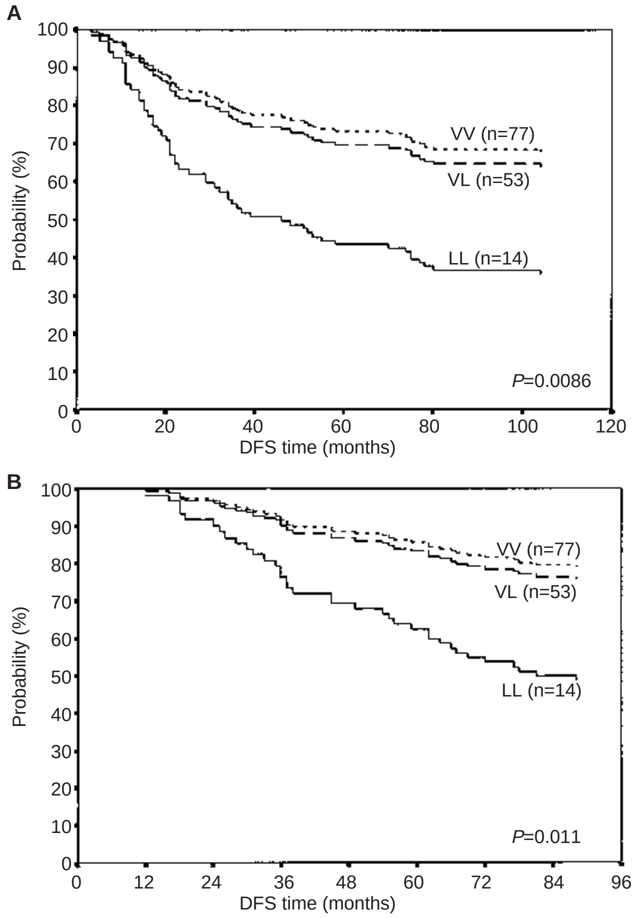

**Figure 3** Disease-free (**A**) and overall survival (**B**) curves in breast cancer patients with VV, VL and LL genotypes. Differences between the 3 genotypes for DFS and OS were determined by log-rank tests

LL allele, respectively, the former having a significantly higher expression. The VL allele showed an intermediate PSA expression which was also significantly higher than that observed in the LL allele. This could be explained by the fact that the GTA genotype encodes for a more active reductase enzyme than the CTA genotype (Kantoff et al, 1997), leading to an increased production of DHT. Higher levels of this androgen up-regulate PSA expression. The VV allele is, for the same reason, related to a higher risk of prostate cancer. PSA is a favourable prognostic factor in breast cancer and patients with elevated concentrations in their tumour extracts, have reduced risk of cancer relapse and death (Yu et al, 1995, 1996, 1998; Griniatsos et al, 1998). Besides PSA, two other proteins expressed in breast tumour tissues, pepsinogen C and pS2, have also been shown to be favourable prognostic indicators (Foekens et al, 1993; Ardavanis et al, 1997; Scorilas et al, 1999, 2000). These proteins are associated with steroid hormone receptor positivity and/or hormonal responsiveness. Our data suggest that the VV alleles, which are related to higher levels of PSA in breast cancer cytosols, are associated with decreased risk in terms of overall survival and relapse (Figure 3).

Studies of prostate cancer have reported that the lengths of the TA polymorphic repeats can influence the 5-α reductase activity. Longer repeats in Caucasians have been linked to down-regulation of the enzyme activity, thereby lowering the DHT production with a resultant decrease in PSA expression and hence lower cancer risk (Kantoff et al, 1997). In contrast, in breast tumours, the longer repeats seem to be associated with enhanced 5-α reductase activity which results in higher PSA expression. It was found that longer TA repeats are associated with breast tumours which have higher PSA content. More specifically, there is a positive relationship between (TA0)/(TA)9 or (TA)9 genotype and PSA levels. Longer TA repeats appear to be favourable prognostic indicators in breast cancer patients (Bharaj et al, 2000). This is supported also by the data in Table 1 which show that 70% of the breast tumours with heterozygous TA(0)/TA(9) repeat lengths are associated with VV alleles, 30% with VL alleles and none with LL alleles. All homozygous TA(9) repeats detected in these tumours are associated with the VV alleles (Table 1). The TA repeats occur in the non-coding region of the SRD5A2 gene and do not affect the function of the resulting protein. However, there is evidence that such TA rich sequences in the 3′ untranslated region of other genes are associated with mRNA instability (Zubiaga et al, 1995). Somatic mutations at the 3′ untranslated region of the SRD5A2 locus which lead to loss of heterozygosity and microsatellite instability of this marker have also been reported (Akalu et al, 1999). Increase in TA lengths may therefore, be associated with relative messenger instability and decreased levels of the 5-α reductase activity. Tumours with longer (homozygous TA(9) and heterozygous TA(0)/TA(9)) repeat lengths, when compared to those with shorter TA(0) alleles by Cox regression analysis, showed a 40% reduction in risk for relapse (Bharaj et al, 2000). This relationship also concurs with the decreased risk observed in the VV alleles (Figure 3) to which the TA(9) repeats appear to be linked (Table 1).

Based on these data, it may be speculated that the VV allele and the longer dinucleotide TA repeat length in breast cancer could be associated with a favourable prognosis and a later onset of the disease (Table 2). This study also reiterates the importance of androgens in the development and progression in breast cancer. Since these 2 polymorphisms are heritable, any resultant effect is present throughout the life. Even small changes in the 5-α reductase activity and the subsequent effect on DHT production can have a significant effect on risk for breast cancer. However, it would be more meaningful if the tissue reductase enzyme activity and DHT levels are measured simultaneously in parallel with these polymorphisms, in order to directly address the biological significance of these polymorphisms. No such data are available to date on breast cancer. Our results raise the possibility that genetic alterations in the 5-α reductase activity may have a role in modifying breast cancer incidence rates, age of onset and aggressiveness of breast cancer. If these findings are confirmed, they may have important implications for breast cancer prevention and possibly treatment. Further studies will be required to further determine the role of androgens in breast cancer pathogenesis and progression.

### REFERENCES

Akalu A, Dimajian DA, Highshaw RA, Nichols PW and Reichardt JK (1999) Somatic mutations at the SRD5A2 locus encoding prostatic steroid 5-alpha reductase during prostate cancer progression. *J Urol* **161**: 1355–1358

Alexiera-Figusch J, van Putten WLS, Blankestein MA, Blonk-Van Der wijst J and Klijn JGM (1988) The prognostic value and relationships of patient characteristics, estrogen and progestin receptors and site of relapse in primary breast cancer. *Cancer* **61**: 758–768

© 2001 Cancer Research Campaign 

Ardavanis A, Gerakini F, Amanatidou A, Scorilas A, Pateras C, Garoufali A, Pissakas G, et al (1997) Relationships between cathepsin D, PS2 protein and hormonal receptors in breast cancer cytosols: Inconsistency with their established prognostic significance. *Anticancer Res* **17**: 3665–3670

Balbin M and Lopez-Otin C (1996) Hormonal regulation of the human pepsinogen C gene in breast cancer cells: Identification of a cis-acting element mediating its induction by androgens, glucocorticoids and progesterone. *J Biol Chem* **271**: 15175–15181

Bertuzzi A, Daidone MG, Di Fronzo G and Silvestrini R (1981) Urinary androgens and tumor estrogen receptor as predictors of ovariectomy response and of survival in advanced breast cancer. *Breast Cancer Res Treat* **1**: 201–205

Bharaj B, Vassilikos EJK and Diamandis EP (1999) Rapid and accurate determination of CAG repeats in the androgen receptor gene using polymerase chain reaction and automated fragment analysis. *Clin Biochem* **32**: 327–332

Bharaj BS, Angelopoulou K and Diamandis EP (1998) Rapid sequencing of the p53 gene with a new automated sequencer. *Clin Chem* **44**: 1397–1403

Bharaj BS, Scorilas A, Maurizia G and Diamandis EP (2000) TA repeat polymorphism of the 5alpha-reductase gene and breast cancer. *Cancer Epidemiology, Biomarkers and Prevention* 9 (4): 387–393

Bloom HJG and Richardson WW (1957) Histological grading and prognosis in breast cancer. *Br J Cancer* **11**: 359–377

Bryan RM, Mercer RJ, Bennett RC, Rennie GC, Lie TH and Morgan FJ (1984) Androgen receptors in breast cancer. *Cancer* **54**: 2436–2440

Coffey DS (1993) Prostate cancer. An overview of an increasing dilemma. *Cancer* **71**: 880–886

Dressler LG, Seamer LC, Owens MA, Clark GM and McGuire WL (1988) DNA flow cytometry and prognostic factors in 1131 frozen breast cancer specimens. *Cancer* **61**: 420–427

Ferguson RA, Yu H, Kalyvas M, Zammit S and Diamandis EP (1996) Ultrasensitive detection of prostate specific antigen by a new time-resolved immunofluorometric assay and the Immulite immunochemiluminescent third generation assay: potential applications in prostate and breast cancers. *Clin Chem* **42**: 675–684

Foekens JA, van Putten WLJ and Portegen H (1993) Prognostic value of pS2 and cathepsin D in 710 human primary breast tumors: Multivariate analysis. *J Clin Oncol* **11**: 899–908

Gormley GJ, Stoner E, Bruskewitz RC, Imperato-McGinley J, Walsh PC, McConnell JD, Andriole GL, et al (1992) The effect of finesteride in men with benign prostate hyperplasia. *N Eng J Med* **327**: 1185–1191

Grattarola R (1973) Androgens in breast cancer. A typical endometrial hyperplasia and breast cancer in married pre-menopausal women. *Am J Obstet Gynecol* **116**: 423–428

Grattarola R, Secreto G, Recchione C and Castellini W (1974) Androgens in breast cancer. II Endrometrial adenocarcinoma and breast cancer in married post menopausal women. *Am J Obstet Gynecol* **118**: 173–181

Griniatsos J, Diamandis E, Gioti J, Karyda I, Vassilopoulos PP and Agnanti N (1998) Correlation of prostate specific antigen immunoactivity (IR-PSA) to other prognostic factors in female breast cancer. *Anticancer Res* **18**: 683–688

Hahnel R and Hahnel E (1996) Expression of the PIP/GCDFP-15 gene and survival in breast cancer. *Virchows Arch* **429**: 365–369

Hall RE, Clements JA, Birrell SN and Tilley WD (1998) Prostate-specific antigen and gross cystic disease fluid protein-15 are co-expressed in androgen receptor-positive breast tumors. *Br J Cancer* **78**: 360–365

Hill, P, Garbaczewski L and Wynder EL (1983) Testosterone in breast fluid. *Lancet* **1**: 761

Kantoff PW, Febbo PG, Giovannucci E, Krithivas K, Dahl DM, Chang G, Hennekens CH, Brown M, et al (1997) A polymorphism of the 5-alpha reductase gene and its association with prostate cancer: A case control analysis. *Cancer Epidemiol Biomarkers Prev* **6**: 189–192

Kaplan EL and Meier P (1958) Nonparametric estimation from incomplete observations. *J Am Stat Assoc* **53**: 457–481

Key TJA and Pike MC (1988) The role of estrogens and progestagens in the epidemiology and prevention of breast cancer. *Eur J Cancer Clin Oncol* **24**: 29–43

Kuenen-Boumeester V, Van der Kwast TH, Claassen CC, Look MP, Liem GS, Klijn JG and Henzen-Logmans SC (1996) The clinical significance of androgen receptors in breast cancer and their relation to histological and cell biological parameters. *Eur J Cancer* **32**: 1560–1565

Labrie F, Sugimoto Y, Luu-The, Simard J, Lachance Y, Bachvarov D, LeBlanc G, et al (1992) Structure of human type II 5 Alpha reductase gene. *Endocrinology* **131**: 1571–1573

Lopez-Otin C and Diamandis EP (1998) Breast and prostate cancer: an analysis of common epidemiological, genetic, and biochemical features. *Endocr Rev* **19**: 365–396

Lowry OH, Rosebrough NJ, Farr AL and Randall RJ (1951) Protein measurement with folin-phenol reagent. *J Biol Chem* **193**: 265–275

Magklara A, Scorilas A, Catalona WJ and Diamandis EP (1999) The combination of human glandular kallikrein and free prostate-specific antigen (PSA) enhances discrimination between prostate cancer and benign prostatic hyperplasia in patients with moderately increased total PSA. *Clin Chem* **45**: 1960–1966

Magklara A, Scorilas A, Stephan C, Kristiansen G, Hauptmann S, Jung K and Diamandis EP (2000). Down-regulation of prostate specific antigen (PSA) and human glandular kallikrein 2 (hK2) in malignant vs non-malignant prostatic tissue. *Urology* **56**: 527–532

Makridakis N, Ross RK, Pike MC, Chang L, Stanczyk FZ, Kolonel LN, Shi CY, et al (1997) A prevalent missense substitution that modulates activity of prostatic steroid 5alpha-reductase. *Cancer Res* **57**: 1020–1022

Makridakis N, Di Salle E and Reichardt JK (2000) Biochemical and pharmacogenetic dissection of human steroid 5alpha-reductase type II. *Pharmacogenetics* **10**: 407–413

Pisani P, Parkin DM, Bray F and Ferlay J (1999). Estimates of the worldwide mortality from 25 cancers in 1990. *Int J Cancer* **83**: 18–29

Recchione C, Venturelli E, Manzari A, Cavalleri A and Martinetti A and Secreto G (1995) Testosterone, dihydrotestosterone and oestradiol levels in postmenopausal breast cancer tissues. *J Steroid Biochem Mol Biol* **56**: 541–546

Reichardt JK, Makridakis N, Henderson BE, Yu MC, Pike MC and Ross RK (1995) Genetic variability of the human SRD5A2 gene: implications for prostate cancer risk. *Cancer Res* **55**: 3973–3975

Reiher A, Kolb R, Reiner G, Jakesz R, Schemper M and Spona J (1987) Prognostic significance of steroid hormone receptor and histopathological characterization of human breast cancer. *J Cancer Res Clin Oncol* **113**: 285–290

Ross RK, Bernstein L, Lobo RA, Shimizu H, Stanczyk FZ, Pike MC and Henderson BE (1992) 5 alpha reductase activity and risk of prostate cancer among Japanese and US whites and black males. *Lancet* **339**: 887–889

Scatchard G (1949) The attraction of proteins for small molecules and ions. *Ann N Y Acad Sci* **51**: 660–672

Scorilas A, Diamandis EP, Levesque MA, Diamandi A, Khosravi MJ, Giai M, Ponzone R, et al (1999a) Immunoenzymatically determined pepsinogen C concentration in breast tumor cytosols: An independent favorable prognostic factor in node-positive patients. *Clin Cancer Res* **5**: 1778–1785

Scorilas A, Trangas T, Yotis J, Pateras C and Talieri M (1999b). Determination of c-myc amplification and overexpression in breast cancer patients. Evaluation of its prognostic value against c-erbB-2, cathepsin-D and clinicopathologic characteristics using univariate and multivariate analysis. *Br J Cancer* **81**: 1385–1391

Scorilas A, Yu H, Soosaipilai A, Gregorakis A and Diamandis EP (2000a). Comparison of the percent free prostate-specific antigen levels in the serum of healthy men and in men with recurrent prostate cancer after radical prostatectomy. *Clin Chim Acta* **292**: 127–138

Scorilas A, Talieri M, Ardavanis A, Courtis N, Yotis J, Dimitriadis E, Tsiapalis C and Trangas T (2000b) Polyadenylate polymerase enzymatic activity in mammary tumor cytosols: A new independent prognostic marker in primary breast cancer. *Cancer Res* **60**: 5427–5433

Secreto G and Zumoff B (1983) Pradoxical effects associated with supra-normal urinary testosterone excretion in pre-menopausal women with breast cancer: increased risk of post mastectomy recurrence and higher remission rate after ovariectomy. *Cancer Res* **43**: 3408–3411

Secreto G, Fariselli G, Bandieramonte G, Recchione C, Dati V and Di Pietro S (1983) Androgen excretion in women with a family history of breast cancer or with epithelial hyperplasia or cancer of the breast. *Eur J Cancer Clin Oncol* **19**: 5–10

Secreto G, Venturelli E, Bucci A, Piromalli D, Fariselli G and Galante E (1996) Intratumor amount of sex steroids in elderly breast cancer patients. An approach to the biological characterization of mammary tumors in the elderly. *J Steroid Biochem Mol Biol* **58**: 557–561

Soreide JA, Lea OA, Varhaug JE, Skarstein A and Kvinnsland S (1992) Androgen receptors in operable breast cancer: relation to other steroid hormone receptors, correlation to prognostic factors and the predictive value for effect of adjuvant tamoxifen treatment. *Eur J Surg Oncol* **18**: 112–118

Spiessl B, Beahrs OH, Hermanek P, Hutter RVP, Scheibe O, Sobin LH and Wagner G (1989) *Illustrated guide to the TNM/pTNM classification of malignant tumors*. TNM atlas, 3rd ed. Berlin; New York: Springer-Verlag, 343 pp

Stoll BA and Secreto G (1992) New hormone related markers of high risk to breast cancer. *Ann Oncol* **3**: 435–438

Vermeulen A, Deslypere JP, Paridaens R, Leclercq G, Roy F and Heuson JC (1996) Aromatase, 17-betahydroxysteroid dehydrogenase and intratissular concentrations in cancerous and normal glandular breast tissue in postmenopausal women. *Eur J Cancer Clin Oncol* **22**: 515–525

 *© 2001 Cancer Research Campaign*

Vilchis F, Hernandez D, Canto P, Mendez JP and Chavez B (1997) Codon 89 polymorphism of the human 5-alpha steroid reductase type II gene. *Clin Genet* **51**: 399–402
Vizoso F, Sanchez LM, Diez-Itza I, Merino AM and Lopez-Otin C (1995) Pepsinogen C is a new prognostic marker in primary breast cancer. *J Clin Oncol* **13**: 54–61
Wigley CW, Prihoda JS, Mowszowicz I, Mendonca BB, New MI, Wilson JD and Russell DW (1994) Natural mutagenesis study of human steroid 5-alpha reductase 2 isozyme. *Biochemistry* **33**: 1265–1270
Wilson JD, Griffin JE and Russell D (1993) Steroid 5-alpha-reductase 2 defiency. *Endocr Rev* **14**: 577–593
Yu H, Giai M, Diamandis EP, Katsaros D, Sutherland DJ, Levesque MA, Roagna R (1995) Prostate specific antigen is a new favorable prognostic indicator for women with breast cancer. *Cancer Res* **55**: 2104–2110
Yu H, Diamandis EP, Levesque MA, Giai M, Roagna R, Ponzone R, et al (1996) Prostate specific antigen in breast cancer, benign breast disease and normal breast tissue. *Breast Cancer Res Treat* **40**: 171–178
Yu H, Levesque MA, Clark GM and Diamandis EP (1998). Prognostic value of prostate specific antigen for women with breast cancer; a large United States cohort study. *Clin Can Res* **4**: 1489–1487
Zarghami N, Grass L and Diamandis EP (1997) Steroid hormone regulation of prostate specific antigen gene expression in breast cancer. *Br J Cancer* **75**: 579–588
Zubiaga AM, Belasco JG and Greenberg ME (1995) The nanomer UUAUUUAUU is the key AU-rich sequence motif that mediates mRNA degradation. *Mol Cell Biol* **15**: 2219–2230

© 2001 Cancer Research Campaign
